# Feasibility and Efficacy of Autologous Bone Marrow Aspirate Transplantation Combined with Human Parathyroid Hormone 1-34 Administration to Treat Osteonecrosis in a Rabbit Model

**DOI:** 10.1155/2017/2484689

**Published:** 2017-03-13

**Authors:** Takeshi Makihara, Tomokazu Yoshioka, Hisashi Sugaya, Katsuya Aoto, Hiroshi Wada, Kenta Uemura, Kenta Tanaka, Hiroshi Akaogi, Masashi Yamazaki, Hajime Mishima

**Affiliations:** ^1^Department of Orthopedics Surgery, Faculty of Medicine, University of Tsukuba, 1-1-1 Tennodai, Tsukuba, Ibaraki 305-8575, Japan; ^2^Division of Regenerative Medicine for Musculoskeletal System, Department of Orthopedics Surgery, Faculty of Medicine, University of Tsukuba, 1-1-1 Tennodai, Tsukuba, Ibaraki 305-8575, Japan

## Abstract

No studies have examined the transplantation of a bone marrow aspirate (BMA) containing mesenchymal stem cells (MSCs) combined with human parathyroid hormone 1-34 (hPTH1-34) administration. Therefore, we evaluated the feasibility and efficacy of autologous BMA transplantation combined with hPHT1-34 administration in a bone necrosis model. The metatarsal bones of rabbits were necrotized using liquid nitrogen, and the rabbits received a BMA transplantation or saline injection followed by hPTH1-34 (30 *μ*g/kg) or saline administration three times per week (*n* = 3-4 per group). The rabbits were euthanized at 12 weeks after the initiation of treatment. No systemic adverse effects or local neoplastic lesions were observed. Importantly, the rabbits in the BMA transplantation plus hPTH1-34 group showed the highest bone volumes and histological scores of new bone. These data confirmed the feasibility of BMA transplantation combined with hPTH1-34, at least during the experimental period. The observed efficacy may be explained by a synergistic effect from the stimulation of MSC differentiation to osteoblasts with hPTH1-34-mediated suppression of apoptosis in osteoblasts. These results indicate the promising potential for BMA transplantation combined with hPTH1-34 administration in bone necrosis treatment. Longer term experiments are needed to confirm the safety of this therapeutic strategy.

## 1. Introduction

Osteonecrosis (ON), which involves the physiological remodeling of bone tissues [1], is thought to be caused by interruptions in blood flow, a decline in the function/number of osteoprogenitor cells, and apoptosis of the osteocytes [2–10]. Under physiological conditions, bone formation and bone resorption are coupled, and the balance between these two processes is maintained appropriately. However, in necrotic lesions, this balance is destroyed, and the mechanical strength of the bone is weakened. ON typically develops in the hip, knee, shoulder, ankle joint, and carpal bones. The collapse and deformity of the necrotized tissue can cause pain or functional impairment and may lead to the development of osteoarthritis, necessitating joint replacement or arthrodesis. However, joint replacements for the hip, knee, shoulder, and ankle are associated with durability issues and the risk of various complications such as infection or thromboembolism. In addition, although arthrodesis for the carpal bones can alleviate pain, this may result in compromised joint function. Therefore, these treatments are not recommended for younger individuals. ON develops frequently in highly active patients when they reach 30–40 years of age as a complication associated with the use of corticosteroids [11]. Thus, it is necessary to develop appropriate treatments for preserving bone that can regenerate necrotized bone and enhance bone strength.

Core decompression [12], autologous bone grafts [13, 14], and bone marrow aspirate (BMA) transplantation [15–18] have been performed in an attempt to regenerate necrotized bone. Core decompression is a method designed to improve blood flow into a necrotized lesion and aid the inflow of precursor cells or growth factors from normal tissues via communication between the necrotized lesion and normal tissues. Autologous bone graft and BMA transplantation simultaneously stimulate bone formation by providing the required components.

The ability to form new bone via BMA transplantation has been confirmed in basic experiments [1] and is thought to depend on mesenchymal stem cells (MSCs) or osteoprogenitor cells. The bone marrow is a source of MSCs, which have the potential to differentiate into mesenchymal tissues such as bone, cartilage, and fat [19]. Autologous MSCs derived from bone marrow have been found to directly differentiate into osteoblasts and contribute to bone regeneration in a rabbit experimental model of ON [20]. Furthermore, a prospective controlled clinical study showed better results in the group receiving bone marrow cell implantation than in that receiving core decompression alone [21]. However, cell therapy is complicated by the lack of mechanical strength at early stages after transplantation. Consequently, patients are at risk of collapse and must avoid mechanical load for a prolonged time. Thus, new strategies to ensure mechanical strength at early stages after transplantation with active osteogenesis at the necrotic site are required.

Human parathyroid hormone (hPTH) 1-34 has been shown to stimulate bone formation and is therefore widely used for the clinical treatment of osteoporosis. Moreover, intermittent administration of hPTH1-34 stimulates the differentiation of osteoprogenitor cells into osteoblasts [22, 23] and suppresses apoptosis in osteoblasts [24], thereby promoting bone formation. Since the BMA contains MSCs or osteoprecursor cells with bone differentiation capability, theoretically, the concomitant use of hPTH 1-34 may stimulate active osteogenesis at an early stage after BMA transplantation. However, the application of such a technique may be limited, as MSCs have been shown to be tumorigenic [25, 26]. In addition, osteosarcomas have been shown to develop in basic experiments with hPTH1-34, and a safety threshold for its use has been suggested [27]. However, to the best of our knowledge, no reports have described the concomitant use of these two methods.

In this study, we evaluated the safety and efficacy of the concomitant use of BMA transplantation and hPTH1-34 administration in the early treatment period using a rabbit model of necrotized small bone [1].

## 2. Materials and Methods

### 2.1. ON Model

Ten 12-week-old Japanese white rabbits (body weight 2.5–3 kg) were used in this study. All protocols involving animals were approved by the institutional review board for animal testing of Tsukuba University. The animals were anesthetized with intramuscular administration of ketamine (50 mg/kg body weight) and xylazine (14 mg/kg body weight). The ON model was established as previously described with confirmation of severe osteonecrotic changes that persisted throughout 20 weeks [1]. Skin incisions of approximately 3 cm were made on the lateral side of the foot, and the fourth metatarsal bone was exposed and carefully removed after the attached ligaments and joint capsule were dissected. The bone was then soaked in liquid nitrogen for 5 min. After the bone was returned to room temperature, a bone tunnel was prepared with a 2 mm drill. An 18-G injection needle was used in the bone tunnel to implant 1 mL bone marrow or saline. The bone was embedded into subcutaneous pockets that had been prepared at two locations on the right cranial posterior and the left caudal posterior sides. The needle was then removed and the skin was sutured.

### 2.2. Preparation of hPTH1-34

hPTH1-34 was synthesized by Asahi Kasei Corporation (Tokyo, Japan). The hormone stock solution was dissolved at 30 *μ*g/mL in physiological saline containing 0.1% rabbit serum albumin.

### 2.3. Experimental Protocol

**Figure 1:**
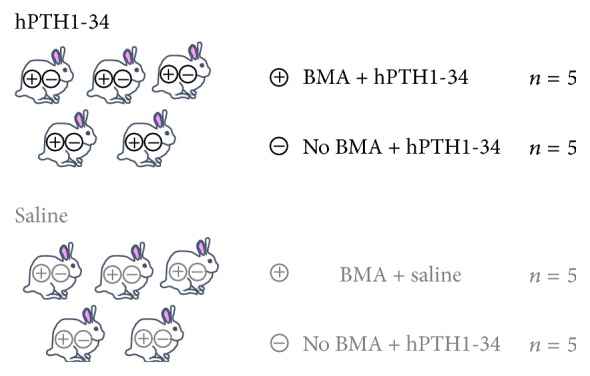
Experimental groups examined in the present study.

The rabbits were divided into two groups, with five rabbits per group. In the first group, rabbits were injected subcutaneously with 30 *μ*g/kg hPTH1-34 three times per week. Rabbits in the second group were injected with saline three times per week. For each rabbit, the metatarsal bones on one side were injected with 1 mL bone marrow collected from the iliac crest; the bones on the other side were injected with saline only (Figure 1). Twelve weeks later, the rabbits were sacrificed via administration of excessive pentobarbital, and samples were collected. Calcein (8 mg/kg) for fluorescent labeling was subcutaneously administered at 2 and 9 days prior to euthanasia.

### 2.4. Safety Evaluation

The body weight of each rabbit was measured once a week. At the start and end of the experiment, 5 mL peripheral blood was collected to measure alkaline phosphatase (ALP), calcium (Ca), and inositol phosphate (IP) levels. The extracted metatarsal bones were fixed with 4% paraformaldehyde and divided into halves, one of which was demineralized with ethylenediaminetetraacetic acid disodium salt for 4 weeks and then embedded in paraffin. A thin slice (5 *μ*m) was taken near the divided surface, and the development of neoplastic lesions was evaluated in samples dyed with hematoxylin and eosin. All samples were evaluated by a pathology expert.

### 2.5. Efficacy Evaluation

Osteocalcin was measured in the collected blood. Before halving the metatarsal bones, microcomputed tomography images were taken (*n* = 3 per group). The data were imported into Mimics (Materialis, Leuven, Belgium), the threshold was set for the computed tomographic value for drawing the bones, and the volume was determined. Histologically, bone formation was qualitatively evaluated from the demineralized samples that were prepared as described above. The other half of the sample was embedded in glycidyl methacrylate to create a nondemineralized sample. A thin slice (5 *μ*m) was taken near the divided surface and dyed with toluidine blue. Calcein labeling was visualized using a fluorescent microscope. Five randomly selected 100x bright and dark fields were captured, and the labeled surface/bone surface ratio (LS/BS) was measured from the two merged images.

### 2.6. Statistical Analysis

Comparisons between groups were carried out by Student's* t*-tests. Differences with *P* values less than 0.05 were considered statistically significant.

## 3. Results

One rabbit that developed an infection and two rabbits whose metatarsal bones were identified only on one side were excluded from further analysis. A total of 14 samples were collected from seven rabbits (BMA plus hPTH1-34: *n* = 3; no BMA plus hPTH1-34: *n* = 3; BMA plus saline: *n* = 4; no BMA plus saline: *n* = 4).

**Figure 2:**
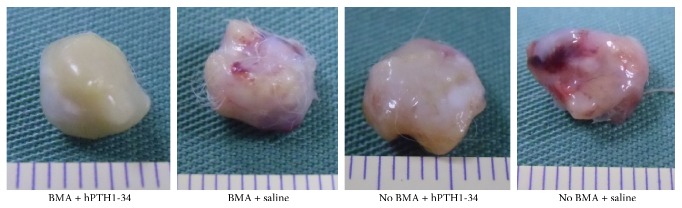
Photomicrograph of an 8 mm long metatarsal bone covered by white fibrous tissue on the surface.

The metatarsal bones that were removed in all groups maintained their original form and were surrounded by white fibrous tissues. No apparent proliferating tissues were observed (Figure 2).

### 3.1. Safety Evaluation

**Figure 3:**
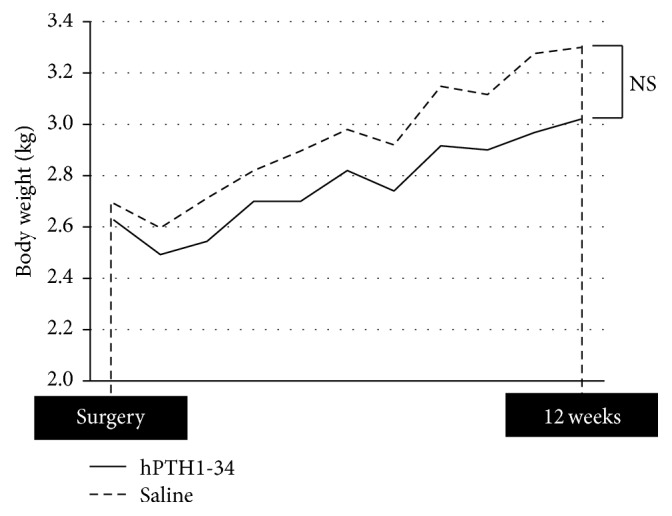
Body weight of rabbits at the time of surgery and at the end of the 12-week experimental period.

The body weight of the rabbits temporarily decreased after surgery in all groups but gradually increased thereafter (Figure 3). No differences in body weights were observed between the groups. In addition, there were no differences in Ca, IP, or ALP levels in the blood (Table 1). Neoplastic lesions were not observed.

### 3.2. Efficacy Evaluation

**Figure 4:**
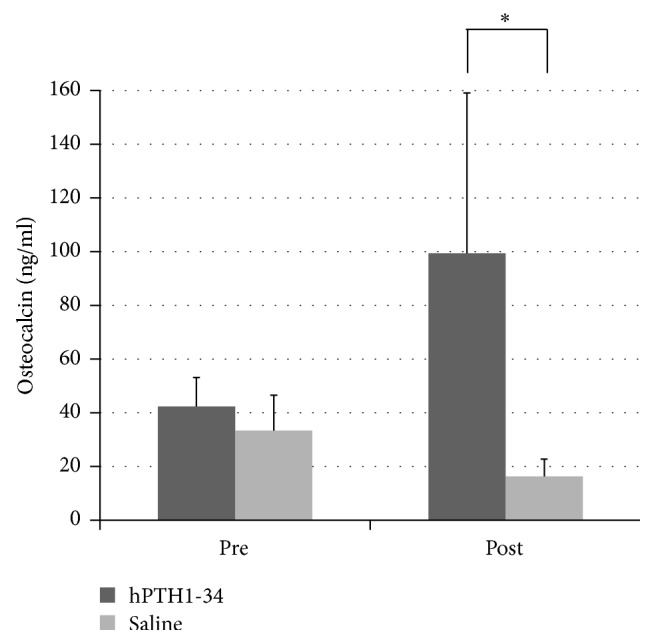
Blood osteocalcin levels. Pre: before surgery; Post: at the end of the 12-week experimental period. ^*∗*^*P* < 0.05.

**Figure 5:**
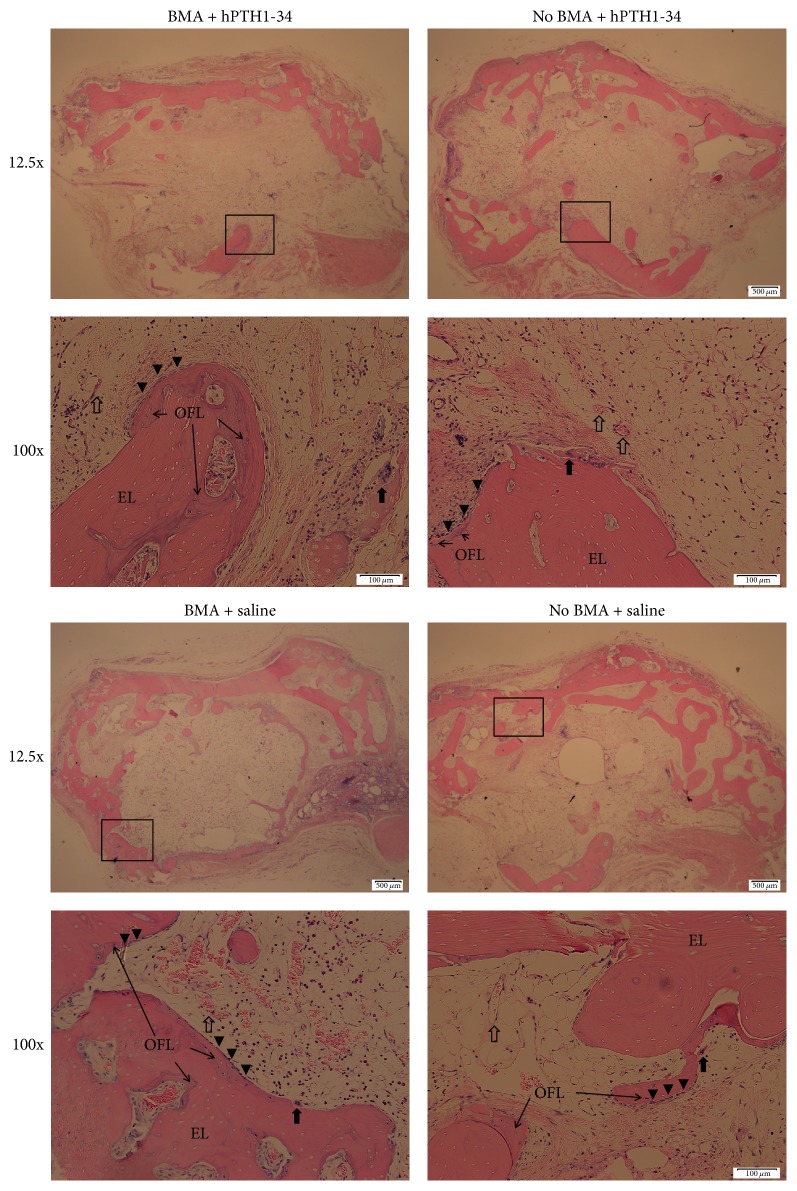
Hematoxylin and eosin staining of bone tissues from all groups (12.5x and 100x). EL, empty lacunae; OFL, osteocyte-filled lacunae. The arrows indicate osteoclasts. The arrowheads indicated osteoblasts. The empty arrows indicate blood vessels.

**Figure 6:**
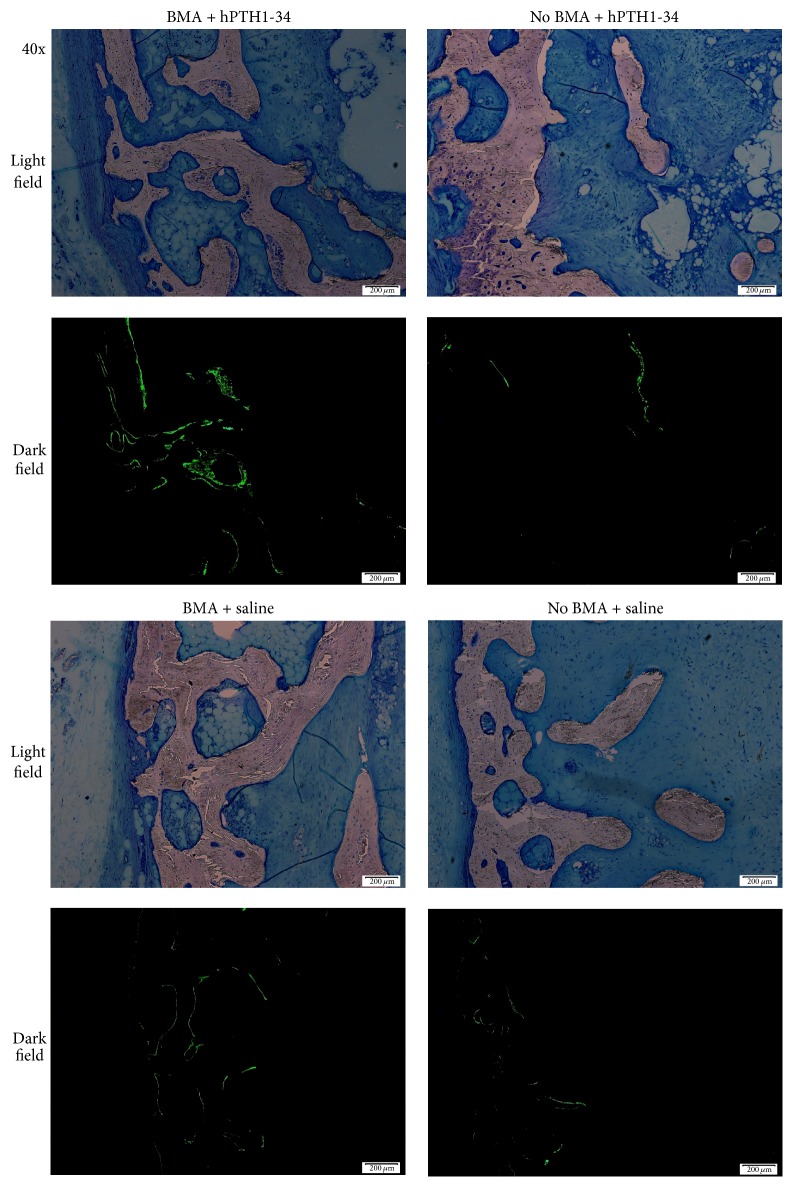
Fluorescent labeling of tissues in each group.

**Figure 7:**
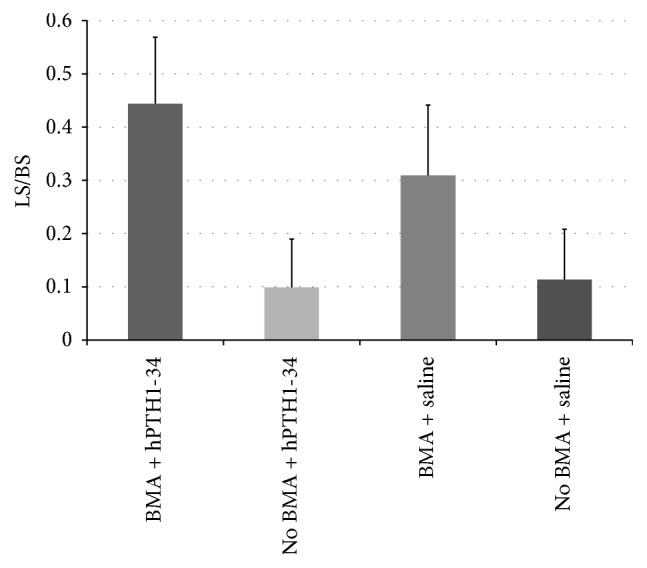
Measurement of the labeled surface/bone surface values.

**Figure 8:**
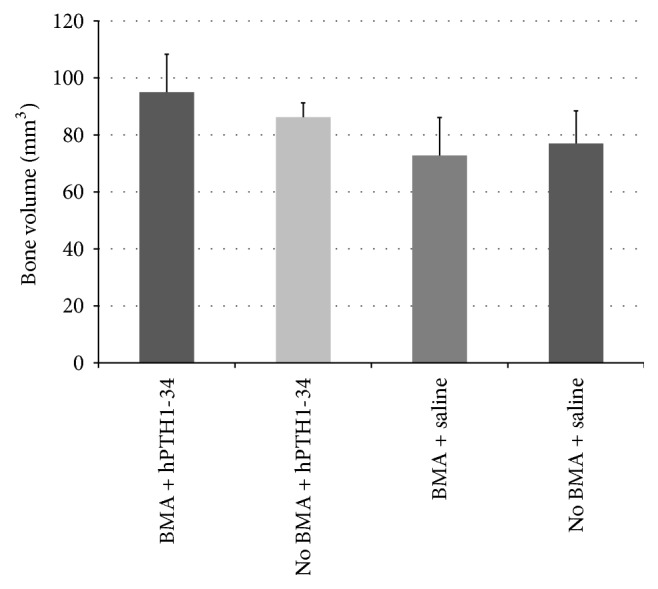
Bone volume measurements by microcomputed tomography.

Preoperative osteocalcin levels did not significantly differ between rabbits treated with hPTH1-34 (42.3 ± 10.8 ng/mL) and those treated with saline (33.4 ± 13.2 ng/mL); however, the osteocalcin levels at the end of the experiment were significantly higher in rabbits treated with hPTH1-34 (99.4 ± 59.7 ng/mL) than in rabbits treated with saline (16.3 ± 6.5 ng/mL, Figure 4). Histological analysis revealed that the metatarsal bones were filled with fibrous tissue in both groups and that blood vessels had formed (Figure 5). Cancellous bones showed a mixture of osteonecrotic changes, including empty lacunae and a fibrous marrow cavity, along with bone tissue consisting of osteocyte-filled lacunae. Arrays of osteoblasts and osteoclast-like cells were observed on the surface of the new bone. In histomorphological analysis, fluorescent labeling was observed in the dark fields in all samples (Figure 6). Both the LS/BS ratios and bone volume measurements were highest in rabbits who received the combination of BMA transplantation and hPTH1-34 (Figures 7 and 8).

## 4. Discussion

In this study, we investigated the safety and efficacy of BMA transplantation combined with hPTH1-34 administration in the early treatment period. Our results demonstrated that the combined treatment was safe during the experimental period, without induction of necrotic lesions, tumors, or weight loss in any of the rabbits. Moreover, bone volume increased in the rabbits treated with BMA transplantation plus hPTH1-34. Therefore, this combination can be considered as a potential treatment for bone regeneration.

Efficacy was confirmed in terms of the systemic drug potency, as indicated by a significant increase in serum osteocalcin levels in the rabbits receiving hPTH1-34. Moreover, in the histomorphological measurements and microcomputed tomography results, numerical values were highest in the rabbits treated with hPTH1-34 and BMA transplantation. Vascularization was observed inside the metatarsal bones. It is possible that hPTH1-34 induced these effects owing to its ability to directly stimulate MSC differentiation [22, 23] and suppress the apoptosis of osteoblasts [24]. However, further studies are needed to examine the specific mechanisms involved.

MSCs have been shown to stimulate the metastatic capacity of malignant cells in animal breast cancer models [28]; however, no reports have described the development of new cancer in noncancerous tissue in response to MSC transplantation. Hernigou et al. [25] conducted magnetic resonance imaging follow-up examinations of 1,873 cases in which MSCs derived from the BMA were transplanted and reported no tumors in the location of the transplantation. Therefore, the use of a BMA containing MSCs may be a safe option for the induction of bone regeneration.

hPTH1-34 is known to stimulate osteogenesis and is widely used in clinical settings. However, during the development phase of the treatment, osteosarcomas have been reported to develop in rats treated with hPTH1-34. Importantly, the incidence of osteosarcoma is reported to be dependent on the dose of the drug and the administration period [27]. Although a safe dose range has been established for human use, the administration period is restricted to 18 months, and administration to patients at risk of developing osteosarcoma is prohibited. In the present study, systemic effects were evaluated by monitoring body weight and blood biochemistry parameters indicative of osteosarcoma, and local effects were assessed via histology. No differences were found in body weight or serum levels of Ca, IP, or ALP, and no adverse systemic effects were observed. The decrease observed in serum IP and ALP levels may have been caused by physiological bone maturation, since only immature rabbits were used in this study. However, there were no significant differences between the hPTH1-34- and saline-treated groups observed in serum ALP levels, an important marker for the diagnosis of osteosarcoma [29, 30]. Moreover, no histological development of neoplastic lesions was noted. These results indicate the feasibility and possible safety of the present treatment, at least in the early period. However, Vahle et al. [31] reported that the formation of bone neoplasms depended on the duration and doses of the treatment and occurred in rats treated for 20 or 24 months. Therefore, longer term experiments are needed to fully investigate the safety of the present treatment.

There are some limitations to this study. First, the number of animals was very small for reliable statistical consideration of efficacy. Second, mechanical evaluations were not performed. Third, certain properties such as the number of MSCs or the variety of cells in the transplanted BMA were not analyzed. Fourth, we could not investigate the state of bone resorption because we did not use any bone resorption markers. Therefore, more detailed studies are needed to determine the optimal number of MSCs, timing of treatment, and interval and frequency of hPTH1-34 administration for achieving proper bone regeneration.

## 5. Conclusion

In our novel ON model, we established a new bone regenerative strategy that involves the synergistic effects from the concomitant use of local BMA transplantation and systemic hPTH administration. The safety and efficacy of this concomitant therapy were confirmed at the early stage of treatment. Although additional studies are still required to validate these findings, these preliminary results show promise of this strategy for clinical applications.

## Figures and Tables

**Table 1:** Blood examination at the start and end of the experiment in groups with and without hPTH1-34 administration.

	hPTH1-34, pre	Saline, pre	hPTH1-34, post	Saline, post
Ca (mg/dL)	12.92 ± 0.19	13 ± 0.32	13.2 ± 0.34	12.9 ± 0.22
IP (mg/dL)	8.2 ± 1.01	7.94 ± 0.49	4.72 ± 0.42	4.92 ± 0.23
ALP (IU/L)	361.8 ± 67.48	441.2 ± 49.38	167.4 ± 24.25	179.2 ± 48.77

ALP, alkaline phosphatase; Ca, calcium; IP, inositol phosphate; post: end of the experiment; pre: at the time of surgery.
